# CHO starter cell lines for manufacturing of proteins with pre-defined glycoprofiles

**DOI:** 10.1186/1753-6561-7-S6-P96

**Published:** 2013-12-04

**Authors:** Karsten Winkler, Michael Thiele, Rita Berthold, Nicole Kirschenbaum, Marco Sczepanski, Henning von Horsten, Susanne Seitz, Norbert Arnold, Axel J Scheidig, Volker Sandig

**Affiliations:** 1 ProBioGen AG, D-13086 Berlin, Germany; 2 Christian-Albrechts-Universität zu Kiel, D-24118 Kiel, Germany; 3 Hochschule für Technik und Wirtschaft Berlin, D-10138 Berlin, Germany

## Backround

Glycosylation of protein therapeutics is influenced by a multifaceted mix of product intrinsic properties, host cell genetics and upstream process parameters. Industrial CHO cell lines may have several deficits in their glycosylation pattern for some applications, like high fucose content (corresponding to a low ADCC profile) and low galactosylation and sialylation levels (proposed to decrease activity and/ or pharmacokinetics).

We have successfully applied the GlymaxX^® ^technology [1] abolishing fucose synthesis in well-established CHO DG44 and K1 platforms and pre-existing producer cell lines (glycan modulator GM1). Here we extend this strategy by other engineering approaches to enable production of protein therapeutics with desired glycosylation features. Through stable integration of other genes for glycosylation enzymes we are able to tune galactosylation (glycan modulator GM2) and sialylation (glycan modulator GM3). These glycan modulators can specifically be combined to address certain desired oligosaccharide patterns.

We postulate that modulating effects of GM2 and GM3 require a specific expression level. In this case the combination of high level target protein expression and defined levels of glycan modulators becomes extremely rare. Therefore, the characterization of clones with individual stable levels of glycanmodulator expression is a prerequisite for industrial application.

## Materials and methods

Two vectors expressing either GM2 alone or GM2 and GM3 in combination were constructed to evaluate modulator effects. This technology was applied to both, CHO-DG44 and K1 cells to generate modified host cell pools. Modulator host cell clones were generated out of appropriate DG44 pools and characterized for growth and modulator gene expression using a 7-day shaker batch culture and RT-qPCR respectively. A human IgG and a Fc-Fusion protein carrying a single N-glycosylation side in the CH2 domain were chosen as model proteins. After stable transfection of human IgG into GM2 and Fc-fusion protein into GM2/3 clones, the resulting test modulator clone pools were analyzed in fed batch shaker assays. Harvested culture supernatants were purified and subjected to N-Glycan profile analysis performed by Hydrophilic-Interaction-Chromatography (HILIC).

## Results

Characterization of modulator host cell clones for proliferation and modulator mRNA expression indicated that growth behavior is not influenced by modulator expression level. Therefore only GMx-mRNA level were used to select five to six clones expressing a broad range of either GM2 alone or GM2 and GM3 in combination. Each selected modulator host cell clone was transfected with the corresponding model protein in duplicates (indicated by A or B).

Final fed batch assays gave typical clone pool results with growth profiles showing high comparability between clone pools expressing the same model protein (Table 1). Peak viable cell densities (VCD) of about 3E7 vc/mL were reached with maximum titers of 1.2 g/L hum IgG and 2.4 g/L Fc-Fusion protein within 12 days, while final viabilities were in most cases above 80%.Up to 3 fold different titers between pools A and B of the same starter clone were observed depending on selection schemes and process management.

**Table 1:** Data of selected clone pools shown in Figure 1.

		Model protein: human IgG							Model protein: Fc-Fusion protein					
				
**Clone pool no**.		1		2		4			6		7		10	
Index		A	B	A	B	A	B		A	B	A	B	A	B
relative modulator mRNA expression														
**GM2**		5.8	5.8	2.5	2.5	0.4	0.4		1.5	1.5	3.7	3.7	0.6	0.6
**GM3**									3.2	3.2	1.4	1.4	0.3	0.3
**Key process parameter**														

**Peak VCD**	**(cell/mL)**	20	20	31	24	28	24		31	24	29	25	27	25
**Final-vitality**	**(%)**	73	82	82	88	87	91		87	87	86	84	89	93
**Titer**	**(g/L)**	0.6	0.4	0.9	0.7	1.0	0.6		2.1	1.0	1.8	1.0	2.3	1.1
**N-Glycan analysis**														

**G0F**	**(%)**	2	1	62	55	71	68		37	24	19	19	46	41
**G1F**	**(%)**	19	13	23	29	18	21		27	32	35	34	33	38
**G2**	**(%)**	3	4	1	1	1	1		1	1	1	1	1	1
**G2F**	**(%)**	61	67	4	6	2	3		3	7	11	10	9	12
**G1FS1**	**(%)**	2	3	<1	<1	<1	<1		9	9	5	7	0	0
**G2FS1**	**(%)**	2	2	<1	<1	<1	<1		19	21	22	11	1	9

GM2 and human IgG expressing clone pools no. 1, 2, 4. Fc-Fusion protein and GM2/3 expressing clone pools no. 6, 7, 10. Duplicates are indicated by A and B. Key process parameter and corresponding results of N-Glycan analysis are shown in the under part of the table.

**Figure 1:**
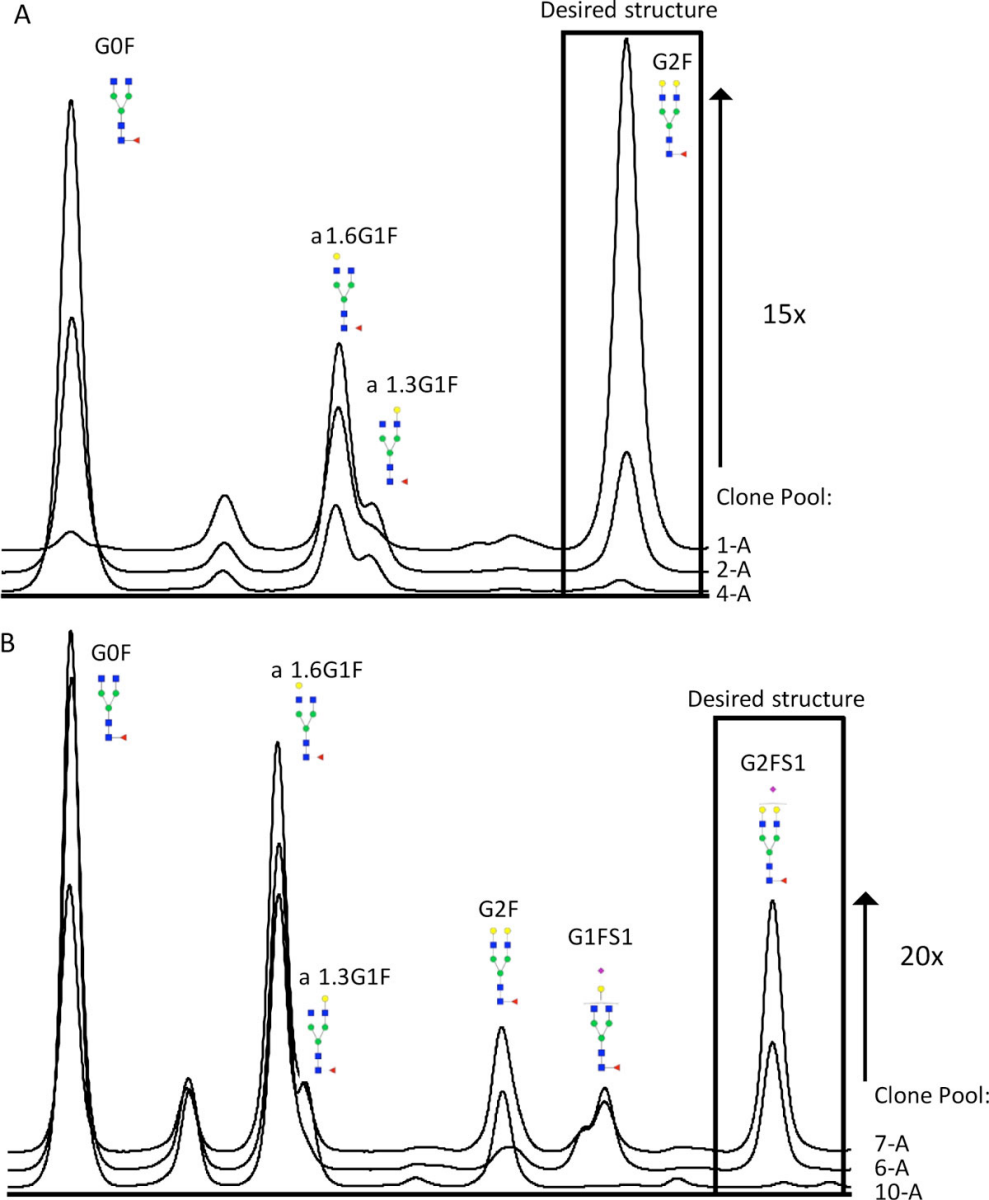
**HILIC chromatograms of clone pools with distinct modulator expression levels**. A: GM2 clone pools, B: GM2/3 clone pools. With increasing GM2 activity a clear shift towards G2F structures can be observed. While the increasing activities of GM2 and GM3 correlates positively with the G2FS1 content.

As it is given by the conveyer like nature of the glycosylation machinery the content of a certain glycan structure cannot be increased without decreasing the output of the preliminary structures. Therefore the hypergalactosylation effect of GM2 should result in a shift towards more G2F structures and for the combination of GM2 and GM3 a shift towards more G2FS1 structures is anticipated, while even the G2F content could be decreased. As shown in Figure 1 the expected shifts were observed, demonstrating that the glycan modulators are working in the intended way. Additionally, we found a positive correlation between the level of modulator gene expression and the degree of glycan modifying effect. Clone pools with highest modulator expression levels displayed the highest content of the desired structures e.g. G2F for GM2 clones and G2FS1 for GM2/3 clones. This reflects a 15 - 20-fold increase of these target structures compared to clone pools with low or moderate modulator expression (Table 1).

Despite substantial differences in productivity and process between A and B clone pool duplicates (2 - 3 fold difference in titers) in most cases only slight shifts of certain oligosaccharide structures were observed (e.g. clone pool 3 - 5 and 8, 9). This indicates that the glycan pattern is more depended on clone specific modulator gene expression than on glycoprotein expression level.

## Conclusions

Expression of GM2 and GM3 in CHO cell lines can effectively change the glycosylation pattern of target proteins in a dose dependent manner. Growth and productivity characteristics are similar to unmodified host cells and maintain their suitability for clinical and commercial production.

The degree of glycomodulation is reproducible and relatively independent of target glycoprotein expression level. This allows a prediction of glycosylation patterns of glyco-proteins produced in certain host cell clones in relation to modulator expression level.

Finally, a comprehensive set of engineered, biopharmaceutical CHO production cell lines were generated and characterized, individually optimized for enhanced ADCC activity, adjusted galactosylation or sialylation levels of the target proteins. This elaborate cellular toolbox allows the rapid and targeted creation of antibody and glycoprotein molecules with specific pre-defined glycan profiles.

## References

[B1] von Horsten HH Ogorek C Blanchard V Demmler C Giese C Winkler K Kaup M Berger M Jordan I Sandig V Production of non-fucosylated antibodies by co-expression of heterologous GDP-6-deoxy-D-lyxo-4-hexulose reductase Glycob 2010 7 1607 1618 10.1093/glycob/cwq109 20639190

